# The Effects of Eye Exercises on Eye-Hand Coordination, Cognitive Functions and Balance Ability of the Elderly: A Randomized Controlled Trial

**DOI:** 10.3390/ijerph22101564

**Published:** 2025-10-14

**Authors:** Onchuma Mueangson, Wanchai Keawmai, Radamanee Pabbumnan, Aisada Chukaithai, Ploynapas Thongdonmuean, Parinya Vongvaivanichakul

**Affiliations:** 1 School of Allied Health Sciences, Walailak University, Nakhon Si Thammarat 80160, Thailand; onchuma.mu@wu.ac.th (O.M.);; 2 Movement Sciences and Exercise Research Center, Walailak University, Nakhon Si Thammarat 80160, Thailand

**Keywords:** gaze stability exercises, eye exercises, elderly, eye-hand coordination, cognitive function, balance

## Abstract

This study investigated the effects of eye exercises on eye-hand coordination, cognitive function, and balance in sixty elderly participants aged 60–70 years who were randomly assigned to an experimental or control group. The experimental group performed Gaze Stability Exercises (GSE) for 50 min per session, while the control group performed eyeball exercises for 10 min. Both groups trained twice a week for four weeks. Assessments of eye-hand coordination, cognitive function, and balance were conducted before and after the intervention. An Analysis of Covariance (ANCOVA), adjusting for baseline scores and gender, was used for between-group comparisons. ANCOVA revealed that the GSE group showed significantly greater improvements in cognitive function and dynamic balance compared to the control group (*p* < 0.05). However, no significant difference was found between the groups for eye-hand coordination. Within-group comparisons showed that both groups improved in eye-hand coordination and cognitive function (*p* < 0.05). These findings suggest that eye exercises, including GSEs and eyeball exercises, can enhance eye-hand coordination and cognitive function in elderly individuals. The dynamic balance improvements observed only in the experimental group highlight the potential of GSEs in balance training. Preliminary findings indicate that consistent eye exercise help improve motor and cognitive skills in the elderly, which requires further study.

## 1. Introduction

As societies worldwide experience rapid aging, ensuring a high quality of life for the elderly has become a critical challenge [1,2]. A primary focus is to maintain independence and slow the physiological declines associated with aging [3]. In this context, falls represent a major concern, often leading to significant morbidity, and even mortality [4]. The causes of falls are recognized as multifactorial, involving both internal and external risk factors [5,6,7]. Beyond just the decline in muscle strength, it is understood that falls often stem from the age-related deterioration of various sensory and neurological systems [8,9]. Crucially, this includes a decline in cognitive and psychomotor abilities that are vital for safe navigation in daily life, such as slower reaction times, impaired decision-making, and reduced eye-hand coordination, all of which are essential for responding to unexpected balance disturbances [10,11,12].

General physical activity is known to reduce fall risk [13,14,15]; recent research has explored more innovative and targeted interventions. For instance, socially engaging group activities such as dancing have been shown to improve not only balance and function but also reduce the frequency of falls by enhancing response speed and coordination [16,17]. In this context, eye exercises, such as Gaze Stability Exercises (GSE), represent another specific intervention aimed at strengthening the visual-vestibular system, which is fundamental to visual-motor control and stability [18,19]. These exercises offer several benefits, including improving balance, enhancing perception and memory, and increasing eye-hand coordination [20,21,22,23,24,25,26].

While eye exercises have been shown to improve specific functions in older adults, a research gap remains concerning their combined effects on eye–hand coordination, cognitive function, and balance. Few studies, particularly randomized controlled trials, have comprehensively evaluated the combined effects of a structured GSE program on these three interrelated abilities simultaneously. Furthermore, it is unclear whether a structured, multi-component program like GSE offers superior benefits compared to simple, unstructured eye movements. Therefore, this study aimed to investigate the effects of a structured GSE program compared to a simple eyeball exercise protocol on eye-hand coordination, cognitive function, and balance ability in older adults.

## 2. Materials and Methods

### 2.1. Subjects

**Figure 1:**
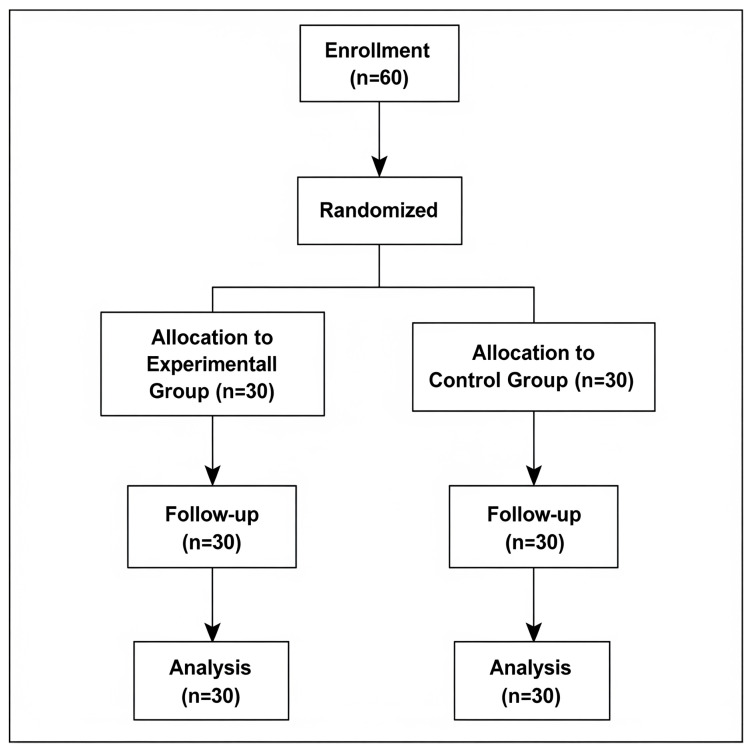
CONSORT flow diagram.

Sixty older adults were recruited through public announcements from community centers for the elderly in Tha Sala District and its surrounding communities, Nakhon Si Thammarat, and were then randomly assigned to either the GSE group (*n* = 30) or the control group (*n* = 30). The flow of participants through the study is illustrated in the CONSORT diagram (Figure 1). The randomization sequence was generated using a computer-based random number generator. Participants were included if they were independent in performing daily activities and capable of walking without assistance. Exclusion criteria included a history of vestibular disorders, neurological conditions, cognitive impairments (Thai Mental State Examination (TMSE) score < 17), musculoskeletal dysfunctions affecting gait, or an inability to stand and walk unassisted. The experimental group participated in structured GSE sessions, while the control group performed only eyeball exercises. Informed consent was obtained before participation, and the study was approved by the Institutional Review Board of Walailak University (WUEC-24-143-01). The trial was registered with the Thai Clinical Trials Registry (Registration No. TCTR20250319005).

### 2.2. GSE Intervention

The GSE protocol followed a structured format adapted from Morimoto et al. (2019) [27] and Park (2017) [20], incorporating six key exercises: eyeball motion range exercise (warm-up), saccadic eye movement, pursuit eye movement, vergence eye movement, vestibular-ocular reflex exercise, and eyeball motion range exercise (cool-down). An attention-control group design was employed. Participants in the control group performed a brief, 10 min protocol of simple eyeball exercises (e.g., slow movements in multiple directions). This protocol was designed specifically to control for the non-specific effects of study participation, such as the regular contact with researchers and the general attention received, rather than to serve as a time-matched equivalent to the GSE intervention.

During GSE sessions, participants focused on a card held at eye level while moving either the target or their head in different directions. Each eye movement exercise lasted 10 s per direction, covering six directions within one minute. Specific components of the GSE program included: Saccadic eye movement: Rapid eye shifts between two stationary targets without head movement. Pursuit eye movement: Tracking a slowly moving target with the eyes while keeping the head still. Vergence eye movement: Moving the head left to right while maintaining focus on a fixed target. Vestibular-ocular reflex exercise: Coordinated head and target movement in opposite directions while tracking with the eyes. Each exercise lasted 5 min, structured as 2 min of exercise, 1 min of rest, followed by another 2 min round. The eyeball motion range exercise was performed for 5 min, structured as 1 min of exercise, 1 min of rest, followed by another round. A full GSE session included 20 min of GSEs, plus 10 min of warm-up and cool-down. Participants in the experimental group performed the exercises twice a week for four weeks in a seated position.

### 2.3. Outcome Measures

(1) All assessments were conducted one week before and one week after the four-week training period by a single outcome assessor who was blinded to the participants’ group allocation. O’Connor Finger Dexterity Apparatus Test:

This test assessed eye-hand coordination by requiring participants to pick up three pegs at a time from a tray of 300 metal pegs and insert them into holes on a metal plate. The number of pegs successfully placed within one minute was recorded.

(2) Picture Completion Test (Healy Pictorial Completion Test I (Healy, 1914))

A measure of perception and memory, this test involved a series of images depicting familiar objects, animals, and individuals with key features missing. Participants were asked to identify the missing elements within 20 s per image.

(3) Timed Up and Go (TUG) Test

This test evaluated balance ability and fall risk in elderly participants. Subjects were instructed to stand from a seated position, walk 3 m, turn around, and return to the chair while being timed. The stopwatch started when the participant began to stand and stopped once they returned to a seated position. Instructions emphasized completing the task as quickly yet safely as possible.

### 2.4. Test Procedures and Data Collection

All assessments were conducted one week before and one week after the four-week training period. Evaluations took place in controlled environments, ensuring consistency across sessions.

### 2.5. Statistical Analysis

All statistical analyses were performed using IBM SPSS Statistics ver. 22.0 (IBM Co., Armonk, NY, USA). Baseline characteristics were compared between groups using the Independent-samples *t*-test, Mann–Whitney U test, or Chi-square test where appropriate. The Paired *t*-test and the Wilcoxon Signed-rank test were used to evaluate pre-post differences within each group. To analyze the difference in variables after the exercise program between the groups, an Analysis of Covariance (ANCOVA) was performed. The model included group and gender as fixed factors, with the respective pre-intervention score serving as a covariate. The level of statistical significance was accepted at a *p*-value < 0.05, and effect sizes were calculated.

## 3. Results

This study divided participants into two groups: an experimental group of 30 individuals who performed GSEs and a control group of 30 individuals who performed eyeball exercises. The baseline characteristics showed no significant differences between the groups, except for gender distribution (*p* = 0.010) (Table 1).

Post-training analysis revealed that the experimental group had significant improvements in eye-hand coordination (*p* = 0.004), perception and memory (*p* = 0.005), and balance ability (*p* = 0.017) (Table 2). Similarly, the control group showed significant improvements in eye-hand coordination (*p* = 0.005) and perception and memory (*p* = 0.007), but not in balance ability (*p* = 0.706).

Furthermore, a comparison of the outcomes between the groups, after adjusting for baseline scores and gender, showed a significantly greater improvement in balance ability for the experimental group compared to the control group (*p* = 0.004). A significant difference favoring the experimental group was also found for perception and memory (*p* = 0.028). However, for eye-hand coordination, no significant difference was observed between the two groups after the intervention (*p* = 0.508). Detailed results, including adjusted mean differences, 95% confidence intervals, and effect sizes, are presented in Table 2.

## 4. Discussion

The findings of this study indicate a significant improvement in eye-hand coordination in both the experimental group, which performed gaze stability exercises, and the control group, which engaged in eyeball exercises. However, when comparing post-intervention, ANCOVA revealed that the GSE program was not significantly more effective than the active-control condition after adjusting for baseline scores and gender. This result may suggest that any form of focused eye movement can provide a foundational benefit for fine motor skills to a certain degree. These results align with the study by Gosewade et al. [22], which demonstrated that gaze stability training enhanced fine motor skills, as evidenced by an increased number of pins placed in the O’Connor Finger Dexterity Test. While the previous study focused on younger participants aged 18–30 years, the present study extends these findings to an elderly population (60–70 years old). The improvement in eye-hand coordination may be attributed to enhanced visuomotor learning resulting from both types of eye exercises, which facilitate faster neural transmission between the eyes and motor muscles. This enhancement leads to improved grasping ability, increased hand movement speed, and better accuracy in placing pegs during the test. Additionally, improved visual focus may contribute to more efficient hand movements and precise targeting of peg placement [28].

This study also found significant improvements in perception and memory in both groups. Consistent with our hypothesis, ANCOVA confirmed that the gaze stability exercise program demonstrated significantly greater efficacy in enhancing perception and memory compared to simple eyeball exercises, even after controlling confounding variables. Participants in the experimental group (gaze stability exercises) and control group (eyeball exercises) exhibited cognitive enhancements consistent with the findings of Roh et al. [21] and Zhang [29], respectively. Furthermore, gaze stability exercises demonstrated greater efficacy in enhancing awareness and memory compared to eyeball exercises, particularly among older adults. While the precise neural mechanisms were not investigated in this study, the observed cognitive benefits are consistent with existing theories. For instance, some literature proposes a potential link between complex bilateral eye movements and enhanced cognitive function, possibly through mechanisms such as interhemispheric interaction. This process plays a crucial role in episodic memory enhancement, which involves the recall of images or events. Consequently, participants in the experimental group demonstrated faster reaction times and higher accuracy in identifying missing elements in the Picture Completion Test [21,30,31]. Notably, while episodic memory improved, semantic memory remained unaffected.

The study further revealed a significant improvement in balance ability within the experimental group following gaze stability exercises, consistent with the findings of Roh et al. [21]. In contrast, the control group, which performed only eyeball exercises, did not show significant changes in balance. It is important to consider not only the statistical significance but also the clinical relevance of these findings. For the Timed Up and Go test, the ANCOVA revealed not only a statistically significant difference (*p* = 0.004) but also a large effect size (ηp^2^ = 0.140). According to established conventions, a large effect size indicates that the GSE program had a substantial impact on performance. An adjusted mean difference of −0.850 s represents a considerable improvement in functional mobility for older adults, which is directly related to daily independence and may imply a meaningful reduction in fall risk. The enhanced balance observed in the experimental group is likely due to the postural stabilization effects of gaze stability exercises, which integrate saccadic eye movements to maintain postural stability. These exercises engage the visual-vestibular system, enabling efficient neural communication between the visual cortex and the parietal-occipital lobe, which plays a key role in gaze stabilization during movement.

During head movements, the transition from saccadic to pursuit eye movements ensures that the central fovea maintains a clear visual field. Additionally, vergence eye movements help refine focus on objects at varying distances, promoting adaptive visual responses to maintain stability while the body is in motion. Prior research has demonstrated that eye exercises can improve vestibular function, leading to better balance control [19,20,21]. A literature review by Park (2017) [20] further supports these findings, indicating that gaze stability exercises significantly improved dynamic balance in older adults, as measured by the Timed Up and Go (TUG) test, compared to eyeball exercises.

Although these findings highlight the benefits of gaze stability exercises for improving motor and cognitive function in elderly individuals, certain limitations should be acknowledged. A primary limitation was the significant gender imbalance between groups that occurred despite randomization. While we statistically controlled for this using ANCOVA, it remains a weakness in design, and future studies should consider stratified randomization to ensure a balanced distribution. Another key limitation is the difference in session duration and complexity between the groups, which introduces a potential confounding effect of time and attention. Furthermore, the study’s small sample size, short 4-week intervention period, and the lack of long-term follow-up restrict the generalizability of the findings and the ability to determine the sustainability of the observed effects. Future research should address these limitations to enhance the applicability of eye exercise programs in older adults.

## 5. Conclusions

This study demonstrates that both structured GSEs and simple eyeball exercises can provide benefits for elderly individuals, particularly for eye-hand coordination and perception. The superior effect of GSEs on dynamic balance suggests that the components of the program are important for specific outcomes. These findings can serve as preliminary data to support the selection of appropriate eye exercises for enhancing motor and cognitive functions in the elderly. However, further investigation in larger, long-term trials is warranted to confirm these benefits and to better understand their potential role in elderly care programs.

## Figures and Tables

**Table 1:** Comparison of baseline data between groups.

	Experimental Group	Control Group	*p*-Value
Age (year) Median (IQR)	62.00 (3.00)	63.00 (3.00)	0.097 ^a^
Gender (Male: Female)	7:23	13:17	0.01 *^c^
Weight (kg) Mean ± SD	57.66 ± 9.24	58.57 ± 7.95	0.684 ^b^
Height (cm) Median (IQR)	155.00 (10.00)	155.00 (15.00)	0.858 ^a^
BMI (kg/m^2^) Mean ± SD	23.32 ± 3.12	23.95 ± 3.37	0.460 ^b^
TMSE (score) Mean ± SD	23.03 ± 2.85	22.53 ± 3.39	0.539 ^b^

^a^ Mann–Whitney U test, ^b^ Independent-sample *t*-test, ^c^ Chi-square, * *p*-value < 0.05.

**Table 2:** Comparison of eye-hand coordination, perception and memory, and balance ability before and after exercise program.

	Experimental Group		Within-Group *p*-Value ^a^	Control Group		Within-Group *p*-Value ^a^	Adjusted Mean Difference [95% CI] ^b^	ANCOVA *p*-Value	Effect Size (ηp^2^) ^c^
	Before	After		Before	After				
O’Connor finger dexterity (pins)	22.5 (9)	28(11)	0.004 **	21 (11)	27.5 (9)	0.005 **	−0.992 [−3.978, 1.994]	0.508	0.008
Picture completion (pictures)	2 (3)	5(1)	0.005 **	2 (2)	3 (2)	0.007 **	1.005 [0.11, 0.899]	0.028 *	0.084
Time up and go test (second)	8 (2)	7(1)	0.017 *	8 (2)	8 (2)	0.706	−0.850 [−1.421, −0.280]	0.004 **	0.140

Data presented as Median (IQR). * *p* < 0.05, ** *p* < 0.01. ^a^ Within-group *p*-value from Wilcoxon Signed-rank test. ^b^ Adjusted mean difference between groups [95% Confidence Interval] from ANCOVA. ^c^ Partial Eta Squared (ηp^2^) as the effect size from ANCOVA.

## Data Availability

The data presented in this study are available on request from the corresponding author. The data are not publicly available due to ethical restrictions concerning participant privacy.

## Abbreviations

GSE	Gaze Stability Exercise
TUG	Time up and go test
TMSE	Thai Mental State Examination
